# The subcellular localization of the hepatitis C virus non-structural protein NS2 is regulated by an ion channel-independent function of the p7 protein

**DOI:** 10.1099/vir.0.027441-0

**Published:** 2011-04

**Authors:** Philip Tedbury, Sarah Welbourn, Arnim Pause, Barnabas King, Stephen Griffin, Mark Harris

**Affiliations:** 1 Institute of Molecular and Cellular Biology, Faculty of Biological Sciences and Astbury Centre for Structural Molecular Biology, University of Leeds, Leeds LS2 9JT, UK; 2 McGill Cancer Centre and Department of Biochemistry, McGill University, Montreal, Quebec, Canada

## Abstract

The hepatitis C virus (HCV) p7 ion channel and non-structural protein 2 (NS2) are both required for efficient assembly and release of nascent virions, yet precisely how these proteins are able to influence this process is unclear. Here, we provide both biochemical and cell biological evidence for a functional interaction between p7 and NS2. We demonstrate that in the context of a genotype 1b subgenomic replicon the localization of NS2 is affected by the presence of an upstream p7 with its cognate signal peptide derived from the C terminus of E2 (SPp7). Immunofluorescence analysis revealed that the presence of SPp7 resulted in the targeting of NS2 to sites closely associated with viral replication complexes. In addition, biochemical analysis demonstrated that, in the presence of SPp7, a significant proportion of NS2 was found in a detergent (Triton X-100)-insoluble fraction, which also contained a marker of detergent resistant rafts. In contrast, in replicons lacking p7, NS2 was entirely detergent soluble and the altered localization was lost. Furthermore, we found that serine 168 within NS2 was required for its localization adjacent to replication complexes, but not for its accumulation in the detergent-insoluble fraction. NS2 physically interacted with NS5A and this interaction was dependent on both p7 and serine 168 within NS2. Mutational and pharmacological analyses demonstrated that these effects were not a consequence of p7 ion channel function, suggesting that p7 possesses an alternative function that may influence the coordination of virus genome replication and particle assembly.

## INTRODUCTION

Hepatitis C virus (HCV) infects over 170 million individuals and is a major cause of chronic liver disease, resulting in cirrhosis and hepatocellular carcinoma (Lavanchy, 1999). Current therapies based on a combination of pegylated alpha interferon and ribavirin are ineffective in around 50 % of the cases due to the high prevalence of genotype 1 resistant strains. This has driven intensive research for the development of virus-specific therapies. Subgenomic replicon (SGR)-based approaches have expedited the development of compounds targeting virus genome replication, but it is only recently, following the development of an infectious culture system for HCV based on the genotype 2a isolate, JFH-1 (Wakita *et al.*, 2005), that it has become possible to study the processes involved in virus assembly.

HCV is a member of the genus *Hepacivirus* of the family *Flaviviridae*. It possesses a positive-sense RNA genome of ∼9.6 kb and forms an enveloped particle ∼60 nm in diameter. The genome contains a single ORF and the translation product is cleaved into 10 mature products by both host and viral proteases. The core (C) and envelope (E1/E2) proteins together with the genomic RNA comprise the infectious virion, whereas the non-structural (NS) proteins NS3–NS5B are both necessary and sufficient for viral genome replication and form membrane associated replication complexes in infected cells (Moradpour *et al.*, 2007). The two remaining proteins, p7 and NS2, are dispensable for HCV RNA replication, but have been shown to play a critical role in the production of infectious virus particles (Jones *et al.*, 2007; Steinmann *et al.*, 2007a).

p7 (63 aa) comprises two *trans*-membrane domains and a conserved basic cytosolic loop (Carrère-Kremer *et al.*, 2002; Lin *et al.*, 1994). It possesses ion channel activity *in vitro* (Griffin *et al.*, 2003; Pavlović *et al.*, 2003; Premkumar *et al.*, 2004) and forms hexa- or heptameric channels (Clarke *et al.*, 2006; Luik *et al.*, 2009), which are blocked by several classes of small molecules (Griffin, 2010). These inhibitors block virion release in cell culture, strongly implicating a functional requirement for ion channel activity during this process (Griffin *et al.*, 2008; Steinmann *et al.*, 2007b). Deletion of p7 or specific point mutations abrogate HCV particle release in culture (Jones *et al.*, 2007; Steinmann *et al.*, 2007a), yet the specificity of these mutations regarding ion channel activity or an as-yet undefined alternative role for p7 has not been established. We recently demonstrated that p7 proton channel function is directly responsible for the enhancement of infectious virion production (Wozniak *et al.*, 2010). Interestingly, p7 point mutants can be rescued by the vATPase inhibitor, bafilomycin A, or by exogenous expression of the influenza M2 proton channel, whereas this is not the case for p7 deletants, suggesting that non-ion channel functions of p7 are disrupted (Wozniak *et al.*, 2010; Brohm *et al.*, 2009).

NS2 possesses a highly hydrophobic N-terminal region (residues 1–93), proposed to contain three *trans*-membrane helices, as well as a C-terminal cytosolic auto-protease domain (residues 94–217) that functions to cleave the NS2–3 precursor (Grakoui *et al.*, 1993). Although NS2 is not required for genome replication, if present (in either NS2 containing SGR or full-length virus) replication is dependent on NS2–3 cleavage (Jones *et al.*, 2007; Welbourn *et al.*, 2005). This is probably due to a requirement for mature NS3, as artificial separation of NS2–3 results in replication-competent genomes and renders HCV independent of mutations that disrupt the NS2 protease active site (Jones *et al.*, 2007). Nevertheless, deletion of NS2, and specifically its C terminus, causes a profound defect in HCV particle production providing clear evidence of a role for mature NS2 in assembly separate to its auto-protease function.

There is increasing genetic and biochemical evidence for cooperation between p7 and NS2 during assembly. Chimeric HCV genomes, where structural proteins from other genotypes are joined to the JFH-1 non-structural region, only efficiently produce particles when the N terminus of NS2 originates from the same sequence as the structural proteins (Pietschmann *et al.*, 2006). Similarly, p7 chimeras were only seen to replicate in chimpanzees when the p7 termini remained parental in origin (Sakai *et al.*, 2003). Several adaptive mutations in both p7 and NS2 have also been described that independently enhance particle production and/or rescue mutations in other regions of the genome (Russell *et al.*, 2008; Yi *et al.*, 2007). Recently, using a recombinant virus expressing a p7–GFP fusion separated from NS2 by an IRES, a physical interaction between p7 and NS2 was demonstrated (Ma *et al.*, 2011). NS2 has also been proposed to interact with other non-structural proteins, providing a potential link between replication and assembly (Dimitrova *et al.*, 2003; Flajolet *et al.*, 2000). Furthermore, p7 and NS2 are generated by an inefficient signallase-mediated cleavage of the E2–p7–NS2 precursor, suggesting an advantage in regulating the amount of mature protein in the cell (Carrère-Kremer *et al.*, 2004). The C-terminal region of p7 has also been proposed to act as a signal peptide for NS2, although both p7 and NS2 are able to associate with endoplasmic reticulum (ER) membranes independently of an upstream signal sequence (Carrère-Kremer *et al.*, 2002; Griffin *et al.*, 2005; Santolini *et al.*, 1995).

Here, we provide evidence for p7-mediated targeting of NS2 to a compartment closely associated with HCV replication complexes, concomitantly causing it to accumulate in raft-like, detergent-insoluble membranes. This effect is independent of p7 ion channel function and could provide a means by which these proteins link HCV replication with the assembly pathway.

## RESULTS

### Characterization of SGRs containing NS2 and p7 sequences

**Fig. 1.**
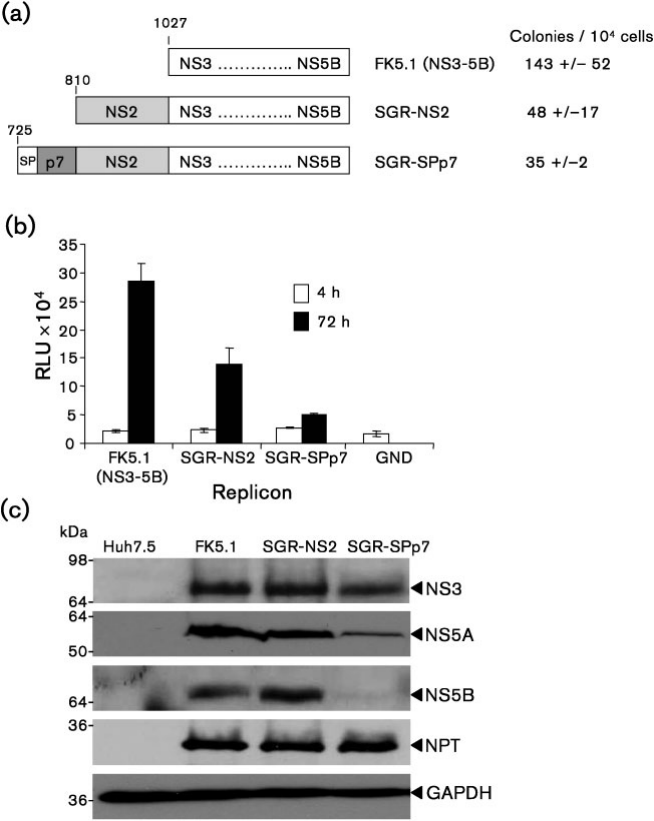
Generation and characterization of p7-containing SGR. (a) Schematic of the constructs produced. Residue numbers are indicated. RNA was generated for each construct and transfected into Huh7.5 cells that were placed under G418 selection for 2 weeks. Colonies were stained with Coomassie brilliant blue and counted. Mean number of colonies±sd is indicated (*n*=3). (b) Huh7.5 cells were electroporated with the indicated SGR RNAs and harvested at 4 and 72 h post-transfection (p.t.) for the determination of luciferase activity. sd are indicated (*n*=3). RLU, Relative light units. (c) Lysates from cells harbouring the indicated SGR were separated by 12 % SDS-PAGE and probed by Western blotting for the indicated proteins.

As discussed, evidence points to an interdependence between p7 and NS2 during HCV assembly. As deletions and/or inactivating point mutations within p7 render full-length JFH-1 genomes non-infectious, we sought to establish an alternative system to analyse the relationship between p7 and NS2. To this end we modified the culture-adapted, genotype 1b, SGR (FK5.1) (Krieger *et al.*, 2001) by adding either NS2 alone (SGR-NS2), or NS2 together with p7 and its E2-derived cognate signal peptide (SGR-SPp7) (Fig. 1a). Both replicons were viable and, as previously demonstrated, addition of NS2 resulted in reduced RNA replication efficiency (Lohmann *et al.*, 1999), measured by both colony formation (Fig. 1a) and luciferase activity (Fig. 1b). The addition of SPp7 gave a further modest reduction in replication efficiency. This may be due to an effect of SPp7 on the stability of the non-structural proteins (Fig. 1c). Intriguingly, replicons containing either p7 in the absence of its cognate signal peptide, or solely the C-terminal helix (comprising the signal peptide required for cleavage between p7 and NS2), were not viable, although this was not due to defects in translation or polyprotein processing (data not shown). We conclude that, at least in the context of a genotype 1b replicon, the presence of an upstream signal peptide is likely to be essential for correct membrane insertion of p7 and subsequent membrane topology of NS2. However, it should be noted that in the J6/JFH1 chimeric virus p7 functionality is retained when preceded by an IRES (E2-IRES-p7 virus) (Jones *et al.*, 2007). Thus, the presence of intact envelope glycoproteins may also facilitate correct membrane insertion of p7 and NS2.

### The presence of p7 results in an altered distribution of NS2

**Fig. 2.**
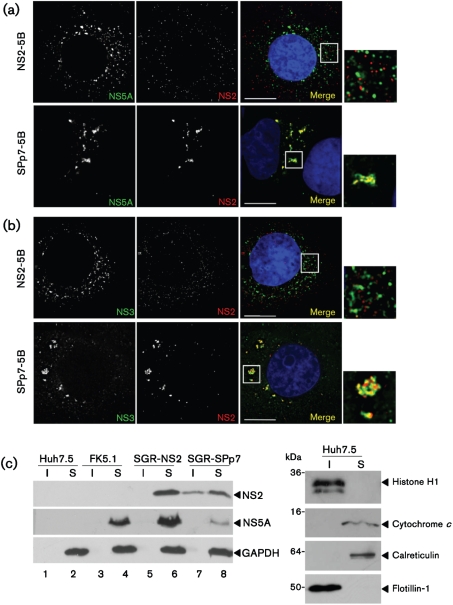
Localization of NS2 in cells harbouring SGRs. SGR harbouring cells were fixed and stained for NS5A (a), or NS3 (b), and NS2, and nuclei were stained with Hoechst 33342. An enlarged area is shown to the right of the merged image, indicated by a box. Bars, 10 μm. (c) SGR-harbouring cells were harvested, lysed and separated into detergent-insoluble and -soluble fractions. Fractions were separated by 12 % SDS-PAGE and probed by Western blotting. The panel on the right shows the distribution of the indicated cellular proteins in the two fractions. I, Detergent-insoluble fraction; S, detergent-soluble fraction.

To gain insight into the potential effects of p7 on NS2 function we established stable Huh7.5 cell lines harbouring SGR-NS2 and SGR-SPp7. The SGR-NS2 harbouring cells expressed the HCV non-structural proteins to similar levels as the FK5.1 (SGR-NS3) cells (Fig. 1c). However, consistent with the differences in replication efficiency (Fig. 1b), in the SGR-SPp7 harbouring cells, non-structural protein expression was lower. Of note, levels of neomycin phosphotransferase were equivalent, suggesting that SPp7 was not having a global inhibitory effect on protein translation from the bicistronic replicon RNA. These observations suggested that p7 was indeed influencing the function of the non-structural proteins, so we therefore examined the subcellular localization of NS2, NS3 and NS5A by immunofluorescence. In cells harbouring SGR-NS2, both NS2 and NS5A exhibited a punctate cytoplasmic localization, but did not colocalize (Fig. 2a). Contrastingly, in cells harbouring SGR-SPp7, NS2 and NS5A were less widely dispersed within the cytoplasm and exhibited a high degree of colocalization, marked by a close apposition and overlap of the NS2 and NS5A signals. This difference was also observed when NS2 and NS3 distribution were analysed – in the SGR-NS2-harbouring cells there was again a lack of colocalization between NS2 and NS3, whilst in the SGR-SPp7-harbouring cells they were less widely dispersed and significantly colocalized (Fig. 2b). These data are consistent with the hypothesis that the presence of p7 directs NS2 to a location proximal to sites of viral genome replication.

We also conducted a biochemical analysis of the distribution of NS2 by lysis in ice-cold Triton X-100 (TX-100) followed by centrifugal separation into detergent-soluble (S) or -insoluble (I) fractions. In the context of the SGR-NS2, NS2 was found solely in the soluble fraction (Fig. 2c, lanes 5 and 6); in SGR-SPp7, however, NS2 was found in both the soluble and insoluble fractions (Fig. 2c, lanes 7 and 8). Western blotting for cellular markers showed that the detergent-insoluble fraction contained raft-like membranes as indicated by the presence of Flotillin-1, as well as nuclear material (histone H1) (Fig. 2c). As NS2 could be clearly shown by immunofluorescence to be absent from the nucleus (Fig. 2a), we conclude that p7 targets at least a proportion of NS2 to a detergent-insoluble fraction. The detergent-soluble fraction contained ER and mitochondria-derived membranes (indicated by calreticulin and cytochrome *c*, respectively) as well as cytosolic proteins (GAPDH). Contrary to previous reports (Gao *et al.*, 2004; Shi *et al.*, 2003), the detergent-insoluble fraction did not contain NS5A (Fig. 2c). This further supports the conclusion that in the presence of p7, NS2 is not being incorporated into replication complexes per se, but rather is being targeted to a location adjacent to these sites of viral genome replication.

### Serine 168 of NS2 is required for the p7-mediated alteration in NS2 distribution, but not for the association with the detergent-insoluble fraction

**Fig. 3.**
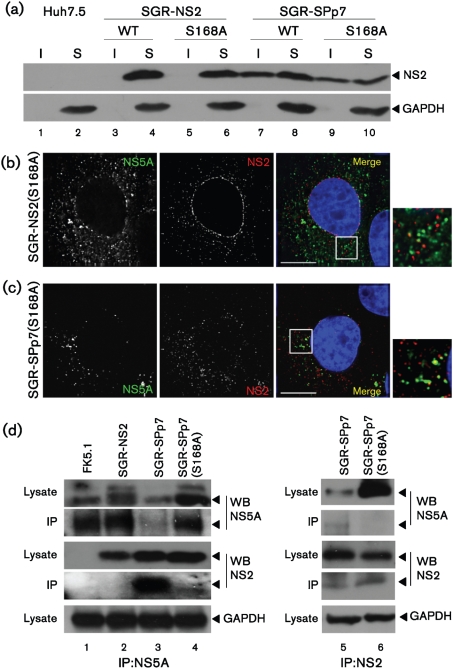
Role of NS2 serine 168 in the p7-mediated altered distribution of NS2. (a) Huh7.5 and SGR harbouring cells were harvested, lysed and separated into detergent-insoluble and -soluble fractions. These were separated by 12 % SDS-PAGE and probed by Western blotting. I, Detergent-insoluble fraction; S, detergent-soluble fraction. (b) SGR-NS2(S168A) and (c) SGR-SPp7(S168A) harbouring cells were fixed and probed for NS5A and NS2, and nuclei were stained with Hoechst 33342. An enlarged area is shown to the right of the merged image, indicated by a box. Bars, 10 μm. (d) SGR harbouring cells were lysed, then immunoprecipitated with either a sheep anti-NS5A serum (left panel), or a rabbit anti-NS2 serum (right panel), and the inputs and eluates probed for NS5A and NS2 using the indicated antibodies. Input lysates were probed for GAPDH as a loading control.

Previous reports have implicated a role for serine 168 within NS2 in virus assembly as a mutation at this residue impairs virion production (Jirasko *et al.*, 2008; Yi *et al.*, 2009), and this residue can be phosphorylated by casein kinase 2 (CK2), leading to proteosomal degradation (Franck *et al.*, 2005). As our data suggested that p7 affected the trafficking of NS2, we therefore asked whether serine 168 also played a role in this aspect of NS2 function. SGR-NS2(S168A) and SGR-SPp7(S168A) replicons were replication competent (data not shown), and could establish stable, replicon harbouring cell lines. In cells harbouring SGR-NS2(S168A), the distribution of NS2 as observed by fractionation (Fig. 3a) or immunofluorescence (Fig. 3b, compare to Fig. 2a, top panel) was unchanged. However, fractionation analysis revealed that in the context of SGR-SPp7, S168A had no effect on the presence of NS2 in the detergent-insoluble fraction (Fig. 3a, compare lanes 7 and 8, and 9 and 10). In contrast, in the context of SGR-SPp7, the S168A mutation resulted in a drastic alteration in the distribution of NS2 such that it closely resembled SGR-NS2 with very little colocalization between NS2 and NS5A (Fig. 3c). These data suggest that serine 168 is not required for the targeting of NS2 to the detergent-insoluble fraction, but it does play a role in the p7-mediated localization of NS2 to sites proximal to NS5A and replication complexes seen in SGR-SPp7 (Fig. 3c).

These data also implied that serine 168 might be involved in a physical interaction between NS2 and components of the replication complexes. To test this we performed a coimmunoprecipitation analysis. As NS5A is a key component of the replication complex and also plays a major role in assembly (Hughes *et al.*, 2009; Masaki *et al.*, 2008; Tellinghuisen *et al.*, 2008), we immunoprecipitated cell lysates with an NS5A antibody and analysed the precipitates by Western blot. NS2 coprecipitated with NS5A from lysates of SGR-SPp7 harbouring cells (Fig. 3d, lane 3), but not from either SGR-NS2 or SGR-SPp7(S168A) lysates (lanes 2 and 4). Furthermore, when lysates were immunoprecipitated with an NS2 antibody, NS5A was only found to precipitate with NS2 in the context of SGR-SPp7 and was absent in the S168A mutant (Fig. 3d, right hand panel). These data are consistent with a direct interaction between NS2 and NS5A, which requires both p7 and serine 168 within NS2. However, we cannot rule out the possibility that this interaction is mediated via additional interactions between NS2, NS5A and other viral or cellular proteins.

### The presence of NS2 in the detergent-insoluble fraction is independent of p7 ion channel activity

**Fig. 4.**
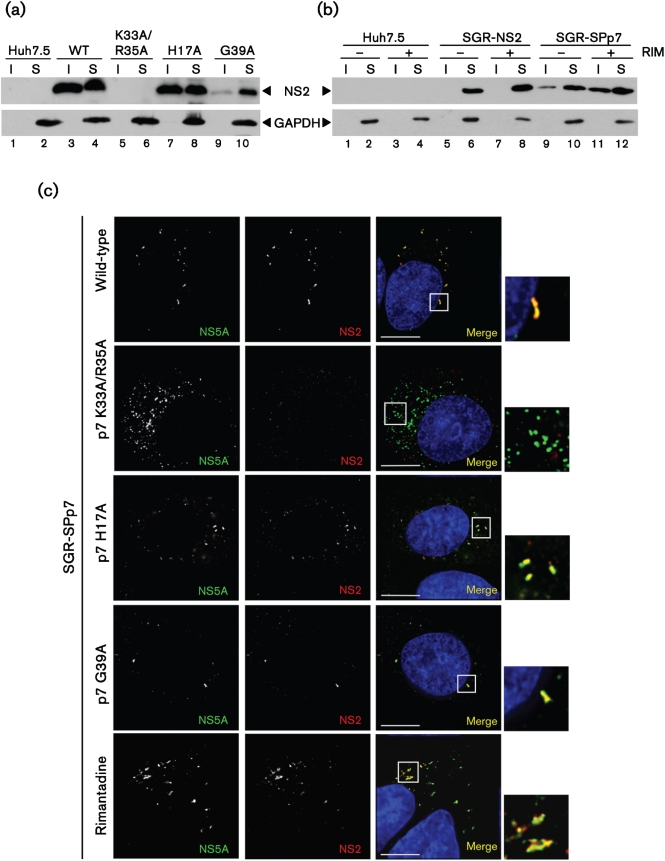
p7 ion channel activity is not required for the altered distribution of NS2. (a) Cells harbouring SGR-SPp7 with mutations in p7 were harvested, lysed and separated into detergent-insoluble and -soluble fractions. Fractions were separated by 12 % SDS-PAGE and probed by Western blotting. (b) Cells were incubated overnight in 50 μM rimantadine then treated as in (a). RIM, Rimantadine; I, detergent-insoluble fraction; S, detergent-soluble fraction. (c) Cells harbouring SGR-SPp7 with the indicated mutations in p7, or SGR-SPp7 wild-type cells treated with rimantadine, were fixed and probed for NS5A and NS2. Nuclei were stained with Hoechst 33342. An enlarged area is shown to the right of the merged image, indicated by a box. Bars, 10 μm.

The ion channel activity of p7 has been shown to be required during virus assembly (Jones *et al.*, 2007; Steinmann *et al.*, 2007a), and it has recently been shown to be critical in protecting intracellular virions from acid pH (Wozniak, *et al.*, 2010). We therefore sought to investigate whether the altered distribution of NS2 required the ion channel function of p7. To this end we disrupted p7 function by introducing specific point mutations into SGR-SPp7, which have previously been demonstrated to disrupt p7 ion channel activity *in vitro* (StGelais *et al.*, 2009) as well as in mammalian cells (Griffin *et al.*, 2004). Mutation of the basic charges on the p7 cytosolic loop (K33A/R35A) has been shown to disrupt both p7 function and secretion of infectious virus (Steinmann *et al.*, 2007a), yet our recent data indicate that it also disrupts polyprotein processing and dramatically reduces the abundance of p7 and NS2 (S. Griffin, data not shown), which may be linked to inefficient membrane insertion observed for this mutant *in vitro* (StGelais *et al.*, 2009). We therefore also introduced two alternative mutations; H17A and G39A, which do not cause such defects in mammalian cells yet abrogate p7 activity *in vitro* without affecting membrane insertion (StGelais *et al.*, 2009). We established stable, replicon harbouring cell lines for each of these mutants and assessed the distribution of NS2 both by fractionation and fluorescence as described above. The effects of mutating the basic loop [SGR-SPp7(KR)] were immediately apparent, with NS2 abundance being reduced below detectable levels by Western blot (Fig. 4a: lanes 5 and 6, Fig. 4c). Consistent with the low abundance of NS2 in Western blots, immunofluorescence revealed an SGR-NS2 type staining pattern for NS5A in cells harbouring SGR-SPp7(KR) and NS2 fluorescence was barely detectable above background (Fig. 4c). The other mutations, however, behaved as the parental SGR-SPp7; NS2 being present in the detergent-insoluble fraction and localizing adjacent to NS5A in bright foci (Fig. 4a: lanes 7–10, Fig. 4c). Interestingly, G39A resulted in a reduction in the amount of NS2 in the insoluble fraction – this is consistent with its more profound effect on ion channel activity (StGelais *et al.*, 2009), perhaps reflective of a more dramatic effect on p7 structure and ion channel-independent functions. Previous *in vitro* studies showed that the H17A defect could be partially overcome by increasing the concentration of purified p7 protein, whereas for G39A this was not the case (StGelais *et al.*, 2009). As an additional control, SGR-SPp7 harbouring cells were treated with 50 μM rimantadine, which inhibits p7 ion channel activity both *in vitro* and in cell culture (Griffin *et al.*, 2008; StGelais *et al.*, 2007; Wozniak *et al.*, 2010); again this did not affect the localization of NS2 (Fig. 4b: lanes 9–12, Fig. 4c). These data confirm that the altered distribution of NS2 by p7 does not require the ion channel activity of the latter protein.

### The p7-mediated distribution of NS2 is observed in the context of the full-length virus

**Fig. 5.**
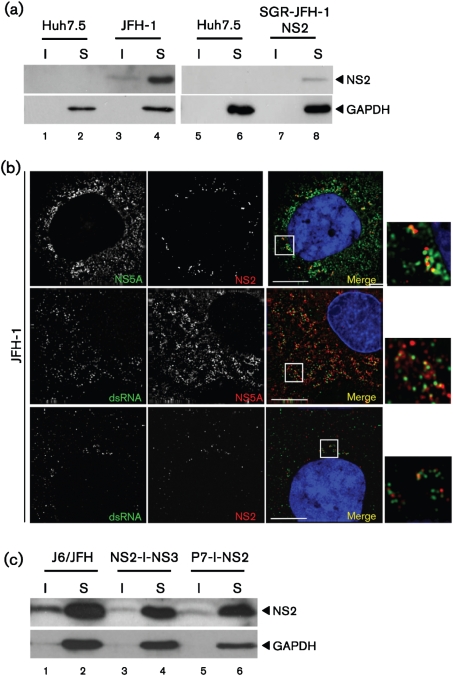
Localization of NS2 in cells infected with JFH-1 or harbouring a JFH-1-derived SGR. (a) Huh7.5 cells transfected with full-length JFH-1 RNA or stably harbouring SGR-JFH-1(NS2-5B) were harvested, fractionated into detergent-insoluble and -soluble fractions, separated by 12 % SDS-PAGE and probed for NS2 or GAPDH. (b) Huh7.5 cells transfected with full-length JFH-1 RNA were fixed at 48 h p.t. in 4 % paraformaldehyde and permeabilized with 0.2 % TX-100 in PBS, prior to staining with antibodies to NS5A, NS2 or dsRNA as indicated. Nuclei were stained with Hoechst 33342. An enlarged area is shown to the right of the merged image, indicated by a box. Bars, 10 μm. (c) Cells were electroporated with J6/JFH chimeric RNAs, wild-type or bicistronic with an IRES between p7 and NS2 (P7-I-NS2) or NS2 and NS3 (NS2-I-NS3) (Jones *et al.*, 2007). Cells were harvested, lysed, separated into detergent-insoluble and -soluble fractions and probed by Western blotting. I, Detergent-insoluble fraction; S, detergent-soluble fraction.

To confirm that our findings were not restricted to the genotype 1b Con1 isolate, the localization of NS2 was examined in the context of the genotype 2a JFH-1 sequence. SGR-JFH-1-NS2 and SGR-JFH-1-SPp7 were produced in an analogous fashion to those described for Con1. Huh7.5 cells stably harbouring these SGRs, or transiently transfected with the full-length infectious JFH-1, were fractionated as previously described. Whereas in the context of full-length infectious JFH-1, NS2 was seen in both the detergent-insoluble and -soluble fractions (Fig. 5a, lanes 3 and 4), in the SGR-JFH-1-NS2 harbouring cells NS2 was found only in the detergent-soluble fraction (lanes 7 and 8), even upon overexposure of the Western blot (data not shown). We were unable to detect NS2 in the SGR-JFH-1-SPp7 harbouring cells as the anti-NS2 serum exhibited reduced sensitivity for genotype 2a NS2 in Western blot, compared with the 1b protein. Additionally, it was not possible to detect NS2 in any of the JFH-1 SGR harbouring cells by immunofluorescence. However, the higher viral protein expression levels in cells transfected with full-length JFH-1 RNA (Fig. 5a) permitted direct detection of NS2 by immunofluorescence (Fig. 5b), and we again confirmed that it localized adjacent to NS5A. The high level of virus replication also permitted the use of the J2 antibody raised to dsRNA. As shown in Fig. 5(b), similar to that observed previously (Targett-Adams *et al.*, 2008), NS5A showed a significant degree of colocalization with dsRNA. This was less apparent for NS2, although NS2 foci were predominantly observed adjacent to sites containing dsRNA (Fig. 5b). This is again consistent with the targeting of NS2 to sites adjacent to replication complexes.

### Targeting of NS2 does not require the p7-NS2 precursor

Although the targeting of NS2 appeared p7-dependent, it was not clear whether this occurred prior to, or following, cleavage of the p7-NS2 precursor. It was therefore necessary to demonstrate whether this precursor mediated NS2 targeting, or whether it occurred via a protein–protein interaction between the mature proteins. We addressed this question by transfecting Huh7.5 cells with RNAs derived from modified chimeric J6/JFH-1-derived genomes in which p7 and NS2 were separated by an IRES [J6/JFH-1(p7-I-NS2)] (Jones *et al.*, 2007). Consistent with the recent observation that NS2 will function *in trans* during HCV particle assembly (Yi *et al.*, 2009), NS2 was targeted to the detergent-insoluble fraction in cells transfected with p7-I-NS2 RNA, as well those transfected with an RNA in which NS2 and NS3 were separated by an IRES (NS2-I-NS3) (Fig. 5c). The data from these experiments confirm that p7 can mediate the altered distribution of NS2 *in* *trans*, and the uncleaved p7-NS2 precursor is not required for this observed effect.

## DISCUSSION

A key unresolved question in HCV biology is how the processes of genome replication and assembly are coordinated and linked, such that infectious virus particles can be generated. In this context, HCV mutants lacking p7 or NS2 are able to undergo genome replication but cannot produce new virions (Jones *et al.*, 2007; Steinmann *et al.*, 2007a); it is likely therefore that both proteins function at the interface between genome replication and packaging. Our data are consistent with the hypothesis that p7 targets NS2 to sites adjacent to replication complexes where it interacts with NS5A and potentially other replication complex components, thereby facilitating the coordination of genome replication and virion assembly.

As well as a redistribution of NS2, biochemical characterization revealed that the presence of the upstream SPp7 sequence resulted in the accumulation of NS2 in a detergent-insoluble fraction. It is unclear whether the distribution of NS2 into detergent-insoluble and -soluble fractions in SGR-SPp7 harbouring cells represents two sites of localization for NS2 or a single site with partial resistance to detergent solubilization. Immunofluorescence data indicate that the latter case is more likely, as in this context the vast majority of NS2 localizes adjacent to replication complexes, i.e. is localized to a single compartment. However, data obtained in the context of the S168A mutation are consistent with the suggestion that the population of NS2 presented in the detergent-insoluble fraction is distinct from that colocalized with replication complexes. Specifically, the accumulation of NS2 in the detergent-insoluble fraction was independent of serine 168, whereas colocalization and interaction with NS5A were dependent on this residue (Fig. 3). The identification of an NS5A mutant that abrogated the NS2–NS5A interaction might help to reconcile this question; however, our data are in agreement with previous work (Franck *et al.*, 2005; Jirasko *et al.*, 2008; Yi *et al.*, 2009), implicating a critical role for serine 168 in the biology of NS2. Intriguingly, cells harbouring SGR-SPp7 exhibited reduced levels of NS5A (and NS3 and NS5B) compared with those harbouring FK5.1 [SGR(NS3-5B)] or SGR-NS2 (Fig. 1c), but the NS5A levels were restored by the S168A mutation (Fig. 3c). This observation suggests that NS2 interacts with NS5A and might ultimately target NS5A for degradation in the absence of ongoing virion morphogenesis. Our data are also consistent with the observation (Yi *et al.*, 2009) that the assembly defects associated with the NS2 S168A mutation can be compensated for by mutations in NS5A. As serine 168 has been shown to be phosphorylated by CK2 *in vitro*, our data suggest that phosphorylation of NS2 may be an important regulatory feature in the ability of NS2 to mediate the interface between genome replication and particle assembly (Franck *et al.*, 2005; Jirasko *et al.*, 2008; Yi *et al.*, 2009). It will be of great interest to establish the effect of p7 on the phosphorylation state of NS2.

The observation that p7 directs NS2 to a site adjacent to replication complexes also provides a potential solution to a paradox of HCV biology, namely that membrane-bound RNA replication complexes isolated from NS3-5B replicon cells are resistant to both nuclease and protease digestion *in vitro* (Aizaki *et al.*, 2004; Yang *et al.*, 2004). How then do nascent genomes get exported from this compartment in order to undergo assembly into virus particles? It may be that NS2 provides such a mechanism by positioning at an exit site of the replication compartment – explaining both the juxtaposition of NS2 and the replication complexes in SGR-SPp7 harbouring cells, and the previously observed requirement for NS2 in the production of infectious virus particles (Jones *et al.*, 2007). Implicit in this hypothesis would be interactions between NS2 and both the structural and non-structural proteins. Consistent with this, it has recently been shown that NS2 interacts with E1, E2, p7, NS3 and NS5A (Ma *et al.*, 2011) and it is likely therefore that NS2 functions to mediate the interaction between the replication complex and the structural proteins, perhaps acting in concert with the recently defined interaction between NS5A and core (Masaki *et al.*, 2008). Alternatively, NS2 may function in the release of RNA from the replication complex and allow its encapsidation into nascent HCV particles. As well as the direct physical interaction, there is genetic evidence for a p7–NS2 interaction from studies of chimeric HCV genomes based on JFH-1. These viruses only produce infectious particles when the N-terminal *trans*-membrane helix of NS2 is derived from the same isolate as the other structural proteins (including p7) (Pietschmann *et al.*, 2006). In addition, our finding that complementing proton channel activity is insufficient to rescue p7 deletants, yet efficiently restores particle production to point mutants confirms that a p7-specific protein–protein interaction is likely to be required for an early stage of virion morphogenesis (Wozniak *et al.*, 2010). The data herein provides additional support for an interaction between p7 and NS2 and furthermore imply that the resultant redistribution of NS2 into close apposition with replication complexes is a critical event during the assembly of infectious virus particles.

## METHODS

### HCV replicon constructs.

An *Rsr*II–*Bsr*GI fragment containing the EMCV IRES and the 5′ end of the NS3 coding region was subcloned from the culture-adapted Con1 NS3–NS5B SGR, FK5.1 (Krieger *et al.*, 2001) into Litmus38. Within this subclone a unique *Nco*I site incorporating the AUG of the HCV ORF was used in conjunction with *Bsr*GI to introduce additional sequences to the N terminus of NS3. HCV-derived sequences were amplified from the genotype 1b Con1 infectious clone using *Pfu* polymerase (Stratagene). *Pme*I–*Bsr*GI fragments of the subclone were transferred back into the FK5.1 backbone. Constructs carrying firefly luciferase were generated by excising the neomycin phosphotransferase coding *Asc*I–*Pme*I fragment and replacing it with the corresponding luciferase coding fragment from pFK5.1luc (Krieger *et al.*, 2001).

To generate JFH-1-derived SGRs, a subclone of the JFH-1 SGR (Kato *et al.*, 2003) was created by cloning the *Pme*I–*Spe*I fragment into a modified Litmus28 vector in which the *Nco*I site was replaced with *Pme*I (Litmus28P). This was linearized with *Nco*I, blunted with mung bean nuclease (New England Biolabs) then digested with *Kas*I. The NS2 coding region of JFH-1 was amplified by PCR using a forward primer containing a start codon and a reverse primer extending to the *Kas*I site in NS3, then digested with *Kas*I and ligated into the subclone. The *Pme*I–*Cla*I fragment of the subclone was replaced in the JFH-1 SGR creating SGR-JFH-1-NS2.

### Mammalian cell culture.

Huh7.5 cells were cultured in Dulbecco's modified Eagle's medium, supplemented with 10 % (v/v) FBS, 100 U penicillin ml^−1^, 100 μg streptomycin ml^−1^, 2 mM l-glutamine and non-essential amino acids (Gibco) at 37 °C, 5 % CO_2_, in a humidified incubator. Stable cell lines were maintained with 500 μg G418 (Melford) ml^−1^. Where indicated rimantadine (provided by GlaxoSmithKline) was prepared as a stock solution (40 mM) in DMSO.

### Transfection of Huh7.5 cells.

Templates for transcription were prepared by linearization with either *Sca*I (FK5.1) or *Xba*I (JFH-1), the latter were also mung bean nuclease treated. RNA was transcribed using Ribomax Express (Promega). RNA was transfected into cells as described previously (Lohmann *et al.*, 1999). Briefly, Huh7.5 cells were trypsinized, washed twice in ice-cold PBS then resuspended in ice-cold PBS at 1 or 2×10^7^ cells ml^−1^ for replicon or virus, respectively. RNA (1 μg for replicons, 10 μg for virus) was mixed with 400 μl cell suspension and electroporated at 270 V and 950 μF. Cells were recovered in pre-warmed medium and seeded as required. Colony formation assays were performed as described previously (McCormick *et al.*, 2004).

### Luciferase assay.

Cells were harvested by the addition of Passive Lysis Buffer (Promega). Luciferase Assay Reagent (Promega) was added (30 μl per 50 μl of cell lysate) and luminescence was measured by using a BMG plate reader.

### Antibodies.

The polyclonal rabbit anti-NS2 serum, 4106, was raised against purified, NS2 cytosolic domain (detailed protocols available upon request). Sheep antisera against HCV non-structural proteins NS3 and NS5A have been described previously (Aoubala *et al.*, 2001; Macdonald *et al.*, 2003), rabbit anti-NS5A serum was provided by Ralf Bartenschlager. Antibodies for cellular proteins were obtained commercially and used as described by the manufacturers; mAbs to calreticulin (Calbiochem), flotillin-1 (BD Biosciences), glyceraldehyde-3 phosphate dehydrogenase (GAPDH; Abcam), and polyclonal sera to cytochrome *c* and histone H1 (Abcam). HRP- (Sigma) or Alexa-Fluor (Invitrogen)-conjugated secondary antibodies were used for Western blotting or immunofluorescence, respectively.

### Cell fractionation.

Cells were harvested, washed twice with PBS, then lysed in GLB [1 % TX-100, 120 mM KCl, 30 mM NaCl, 5 mM MgCl_2_, 10 % glycerol (v/v) and 10 mM PIPES-NaOH, pH 7.2], supplemented with Complete protease inhibitor cocktail (Roche), on ice for 15 min. Insoluble material was pelleted by centrifugation at 500 ***g*** for 5 min at 4 °C. Lysates were clarified by a second centrifugation at 500 ***g***, and the pellets were washed twice in GLB. Normalized lysate and pellet fractions were analysed by Western blotting.

### Immunoprecipitation.

Cells were harvested and lysed in IP buffer [20 mM Tris/HCl pH 7.4, 135 mM NaCl, 1 % TX-100, 0.5 % sodium deoxycholate and 10 % glycerol (v/v)], supplemented with Complete protease inhibitor cocktail (Roche). Lysates were diluted to 2 mg total protein ml^−1^ then 500 μg protein was mixed with 2 μl antiserum at 4 °C. Following overnight incubation, 25 μl magnetic protein G beads (New England Biolabs) were added and the samples incubated for 4 h at 4 °C. Beads were washed three times in IP buffer then eluted by boiling in 2× Laemmli buffer for 5 min.

### Indirect immunofluorescence microscopy.

Cells, seeded onto coverslips 24 h prior to fixation, were washed twice with PBS then fixed with 4 % paraformaldehyde for 10 min at room temperature. Following washing, cells were permeabilized with 0.2 % TX-100 in PBS for a further 10 min, washed twice with PBS, then incubated with primary antibody diluted in PBS/10 % FBS in for 1 h at room temperature. The coverslips were washed with PBS/10 % FBS then incubated with Alexa-Fluor-conjugated secondary antibody. The coverslips were then stained with a second primary antibody, if required, by the same method. Before mounting onto slides, the coverslips were incubated with Hoechst 33342 (Molecular Probes) diluted 1 : 10 000 in PBS for 5 min to stain nuclei, then washed twice in PBS. Coverslips were mounted with Citifluor AF1 (Agar Scientific). Images were captured using an Olympus IX71 microscope with a ×100 oil immersion objective with a numerical aperture of 1.35 (DeltaVision – Applied Precision). *Z*-stacks were collected comprising optical slices of 0.2 μm and deconvolved by using Softworx software (Applied Precision).
